# Light-Controlled Functions with Metal–Organic Capsules: From Guest Release to Catalysis, Separation, and Molecular Transport

**DOI:** 10.1021/acs.accounts.5c00790

**Published:** 2026-01-02

**Authors:** Amit Ghosh, Jonathan R. Nitschke

**Affiliations:** Yusuf Hamied Department of Chemistry, 2152 University of Cambridge, Lensfield Road, Cambridge CB2 1EW, United Kingdom

## Abstract

Light offers a clean and precise means to control chemical processes. These advantages have opened the door to the development of dynamic host–guest systems, whose functions can be turned on or off with specific wavelengths. Over recent years, we have developed a suite of light-responsive metal–organic capsules that use azobenzene photoisomerization to direct functions that include reversible guest encapsulation, selective molecular separations, controlled catalysis, and directional mass transport. These capsules, assembled via subcomponent self-assembly, incorporate azobenzene-based ligands that undergo photoinduced *trans*–*cis* isomerization. This reversible switching induces cage disassembly or changes in host–guest binding, enabling light to act as an external signal to modulate activity.

In this Account, we summarize five key studies that trace the evolution of this platform, from basic molecular recognition and guest release to complex, multicomponent systems capable of energy transduction and spatial molecular control. We describe (i) the design and mechanistic studies of phototriggered guest release using a tetrahedral Zn_4_L_4_ cage; (ii) the use of an architecture built on this initial work to purify progesterone selectively from mixed steroidal systems; (iii) light-gated catalytic activation using a caged perrhenate system; (iv) selective lithium ion extraction using photoswitchable sandwich architecture; and (v) a Maxwell’s Demon-inspired setup that achieves directional molecular pumping across centimeter-scale distances. Collectively, these studies demonstrate how light-responsive metal–organic capsules can be programmed to perform diverse chemical functions, including guest release, selective separations, catalysis, ion extraction, and directional transport. This body of work establishes a platform for the future development of integrated, autonomous, and energy-efficient light-driven supramolecular technologies.

## Graphical abstract

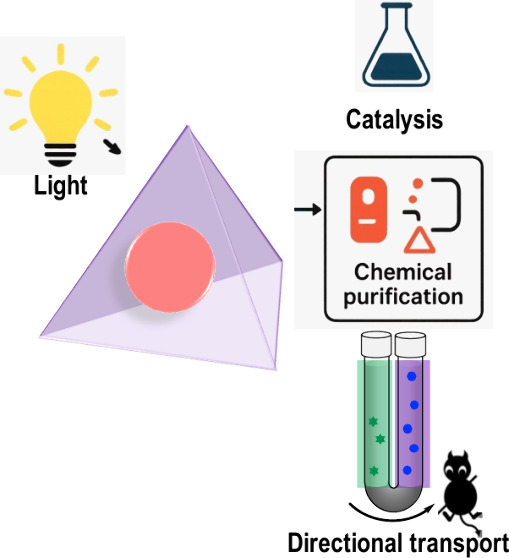

## Key References

-

Ghosh, A. ; Slappendel, L. ; Nguyen, B–N. T. ; von Krbek, L. K. S. ; Ronson, T. K. ; Castilla, A. M. ; Nitschke, J. R. Light-Powered Reversible Guest Release and Uptake from Zn_4_L_4_ Capsules. J. Am. Chem. Soc. 2023, 145, 3828–3832 36753330 10.1021/jacs.2c10084PMC9951218.^1^ A tetrahedral Zn_4_L_4_ capsule incorporating azobenzene ligands undergoes light-triggered disassembly and reassembly, enabling reversible release and uptake of an anionic guest under UV and visible light.
-

Ghosh, A. ; Pruchyathamkorn, J. ; Espinosa, C. F. ; Nitschke, J. R. Light-Driven Purification of Progesterone from Steroid Mixtures Using a Photoresponsive Metal–Organic Capsule. J. Am. Chem. Soc. 2024, 146, 2568–2573.38230667 10.1021/jacs.3c11005PMC10835723 ^2^ A photoresponsive Zn_4_L_4_ capsule selectively binds progesterone from mixed steroid solutions and releases it upon irradiation, enabling light-controlled steroid separation under mild conditions.
-

Ghosh, A. ; Thoburn, J. D. ; Nitschke, J. R. Light-Responsive Aldehyde-Reduction Catalysis Through Catalyst Encapsulation. Angew. Chem. Int. Ed. 2024, e202419575.10.1002/anie.20241957539530278 ^3^ A perrhenate catalyst encapsulated in a light-sensitive capsule is reversibly released by irradiation, allowing temporal control of aldehyde hydrosilylation via cage disassembly and reassembly.
-

Du, Y. ; Ghosh, A. ; Teeuwen, P. C. P. ; Wales, D. J. ; Nitschke, J. R. Light-Driven Lithium Extraction from Mixtures of Alkali Cations Using an Azobipyridine Ligand. J. Am. Chem. Soc. 2025, 147, 20205–20211.40489699 10.1021/jacs.5c05885PMC12186520 ^4^ A visible-light-responsive metal–organic architecture selectively extracts lithium ions from mixtures of alkali metal ions, offering a solar-energy powered route to critical element separation via azobipyridine switching.
-

Pruchyathamkorn, J. ; Nguyen, B.-N.T. ; Grommet, A.B. ; Novoveska, M. ; Ronson, T. K. ; Thoburn, J. D. ; Nitschke, J. R. Harnessing Maxwell’s Demon to Establish a Macroscale Concentration Gradient. Nat. Chem. 2024, 16, 1558–1564.38858517 10.1038/s41557-024-01549-2PMC11374679 ^5^ A coordination cage selectively binds the *trans*-isomer of tetrafluoroazobenzene, driving directional mass transport across a liquid membrane under light control, mimicking Maxwell’s demon by pumping a cargo unidirectionally.

## Introduction

Stimuli-responsive supramolecular systems have emerged as valuable tools for regulating chemical processes through external control.^6,7^ Light is a particularly attractive stimulus due to its clean, reversible, and spatiotemporally precise applicability.^8^ When used to modulate host–guest interactions,^9^ catalysis,^10^ and transport in coordination-based architectures,^11^ light enables the regulation of function with high selectivity and minimal environmental impact.^12,13^ Subcomponent self-assembly provides an efficient route to construct dynamic metal–organic capsules with built-in responsiveness.^14^ This method relies on the simultaneous formation of reversible metal–ligand bonds and imine (C=N) condensation to generate discrete polynuclear structures.^15–18^ By incorporating azobenzene building blocks into subcomponents, we have developed tetrahedral Zn_4_L_4_, Cd_4_L_4_ and Fe_4_L_6_ capsules that respond to light via the *trans*–*cis* isomerization of their azobenzene moieties. The structural transition between the two isomeric states alters the geometry and stability of the overall cage, which in turn modulates guest encapsulation, reactivity, or transport.

The modularity of this design has allowed us to investigate a broad scope of light-gated chemical behavior, ranging from reversible guest release and molecular separation to catalytic control, ion recognition, and directional molecular motion. Our systems operate in solution under mild conditions and can be reset or tuned using different wavelengths of light. This Account describes how we have applied light-responsive cage architectures across five distinct projects. These examples illustrate the functional diversity that arises from integrating photoresponsive ligands into self-assembled structures and highlight new directions in the development of light-driven chemical systems.^19–21^

## Design Principles of Light-Responsive Capsules

Capsules can be designed to be photoresponsive through the incorporation of azobenzene moieties within their ligand frameworks.^22–28^ In their extended *trans* form, these moieties promote the formation of rigid architectures via subcomponent self-assembly. Upon UV irradiation, *trans*–*cis* isomerization introduces a bend that distorts the coordination environment of a proximate metal center, leading to cage opening or disassembly (Figure 1). Visible light reverses this transformation, restoring the *trans* form and promoting reassembly.

**Figure 1:**
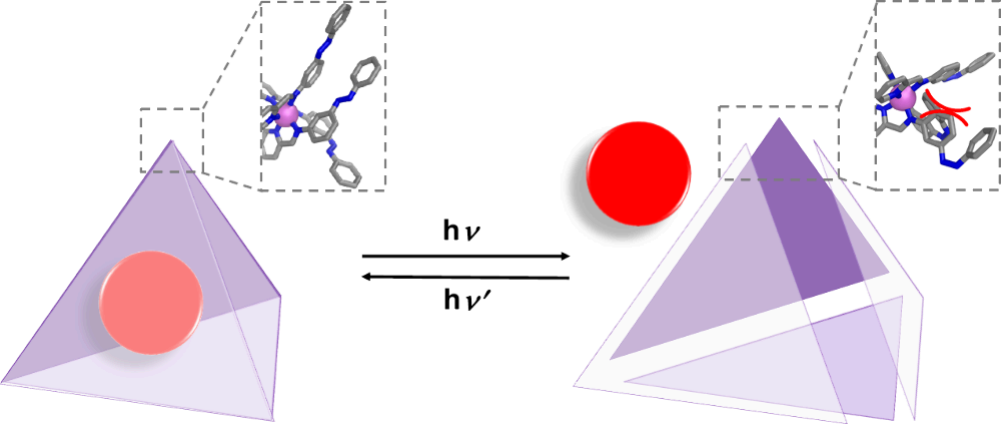
Guest release from a tetrahedral capsule. UV irradiation induces *cis* isomerization of azobenzene ligands, destabilizing the cage and releasing the guest. Visible light restores the *trans* form and re-encapsulates the guest.

In an alternative strategy, azobenzene units are positioned within the metal-coordination motif itself.^29^ Here, isomerization affects metal–ligand binding strength directly: the *cis*-to-*trans* switch generates a geometry unsuitable for coordination, which can lead to metal ion release. Key design considerations include the positioning of azobenzene switches to effectively transmit the steric effects of structural change, the use of metal centers tolerant to geometric perturbation, and ligands that maintain switching fidelity over multiple cycles. Although not yet realized in our current systems, modification of the substitution pattern on the azobenzene core represents a promising route to tuning absorption wavelengths and photoisomerization behavior in future designs.^30–32^ Together, these features define a modular strategy for constructing supramolecular systems whose structure and function can be reversibly controlled using light.

### Light-Induced Guest Release via Cage Disassembly

Although a few self-assembled metal–organic architectures had previously exhibited light-triggered disassembly and reassembly, photoswitchable cages capable of reversible guest release upon irradiation have remained rare.^33–37^ The Zn_4_L_4_ capsule **1** reported in 2023 represents the first example of a tetrahedral cage capable of reversible, light-controlled guest release and uptake (Figure 2a).^1^ Capsule **1**, and related capsules described in this Account, were characterized by ^1^H NMR, ^1^H–^1^H COSY, DOSY, and ESI-MS, with single-crystal X-ray diffraction analysis undertaken for an analogue; all measurements are consistent with the assigned structures as opposed to alternatives. Its tetrahedral framework was obtained by subcomponent self-assembly from azobenzene-derived amines, tritopic formylpyridines, and Zn^II^ ions. In this structure, the azobenzene groups are positioned at the cage vertices and project outward from the framework. Their *trans* configuration is linear, allowing the overall architecture to remain stable, with minimal steric interaction between ligands. Under these conditions, **1** encapsulates its counteranion, bis­(trifluoromethanesulfonyl)­imide (Tf_2_N^–^), within the central cavity. Azobenzene subcomponents thermally favor the *trans* conformation; UV irradiation produces up to 71% *cis*, which relaxes back slowly (*t*_1/2_ = 46 min at 75 °C), persisting long enough to influence cage structure.

**Figure 2:**
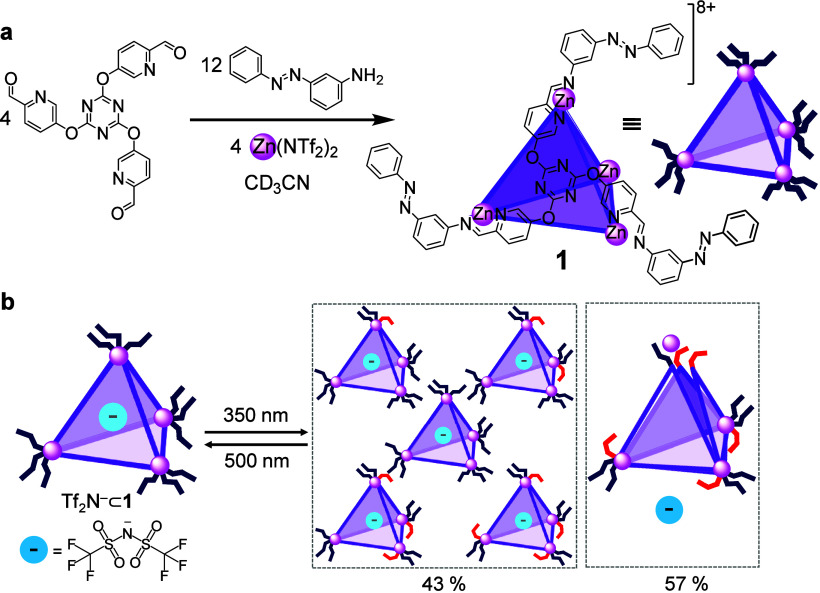
(a) Schematic of the synthesis of Zn_4_L_4_ cage **1** and its cartoon representation. (b) Cartoon showing the photoswitching of **1**, with the cage opening after the fifth azobenzene switches, and its five guest-binding states. *Trans-* and *cis-*azobenzene units are depicted in blue and red, respectively.

Irradiation at 350 nm converts azobenzene units into the *cis* configuration. The bends of the *cis* isomers introduce steric congestion at the cage vertices, progressively weakening Zn–imine coordination. As the proportion of *cis* increases, the framework loses stability and disassembles, releasing the encapsulated Tf_2_N^–^ guest into solution (Figure 2b). Here, “disassembly” refers to the loss of Zn–imine coordination bonds and the partial opening of the tetrahedral framework, as evidenced by the appearance of free subcomponents and Tf_2_N^–^ in the NMR spectra. Illumination at 500 nm restores the azobenzene groups to their *trans* configuration, relieving steric strain and driving reassembly of the tetrahedral cage, which then re-encapsulates the guest. Similar light-triggered release and recapture were observed for other weakly coordinating anions such as PF_6_^–^, TfO^–^, and BF_4_^–^. In related experiments, we observed that azobenzene units positioned *ortho* or *para* to the aniline subunit had minimal influence on guest release under light irradiation, compared to *meta*-substituted azobenzenes. The reversible switching process operates cleanly under ambient conditions and can be repeated over multiple cycles without fatigue. This study demonstrated that peripheral photoresponsive ligands can control the structural integrity of an M_4_L_4_ tetrahedral metal–organic capsule, laying foundations for later applications in molecular separation and catalysis.

### Mechanistic Insights into Guest Release

The mechanism of guest release was investigated directly using ^19^F NMR spectroscopy of the encapsulated Tf_2_N^–^ anion. In the dark, two sharp resonances were observed in a 1:7 ratio, corresponding to encapsulated and free Tf_2_N^–^, respectively. Upon irradiation at 350 nm for 10 min to reach the photostationary state, the intensity of the encapsulated signal decreased by approximately 57%, indicating partial guest release into solution, which contributed to the free-anion resonance. Most notably, the remaining 43% of encapsulated signal resolved into five distinct peaks. These were assigned to capsules containing between zero and four *cis*-azobenzene moieties, with each cage able to tolerate at most one *cis* unit per vertex, before the capsule opened to release its guest (Figure 2b). Thermal reversion experiments supported this assignment: when the irradiated mixture was heated at 75 °C, the five encapsulated signals converted progressively back to the all-*trans* species, consistent with sequential *cis*-to-*trans* isomerization and progressive cage reassembly.

This mechanistic picture highlights how a cooperative threshold, defined by the critical fraction of *cis*-isomerized ligands required to destabilize the framework, governs disassembly. Insights thus gained suggest strategies for tuning responsiveness in future designs: By controlling the number and placement of photoswitches, modifying the mechanical tolerance of metal–ligand junctions, or varying guest affinity, it should be possible to modulate the release threshold and kinetics.

## Chemical Purification Using Photoresponsive Metal–Organic Architectures

Efficient chemical separation remains a central challenge in both industrial and environmental chemistry, often demanding energy-intensive, solvent-heavy methods.^38–40^ Photoresponsive metal–organic architectures offer a promising alternative, by coupling molecular recognition with externally controllable release. Through judicious design of azobenzene-based ligands, light can be used to regulate the stability, permeability, and guest affinity of coordination cages, enabling on-demand capture and liberation of target species under mild conditions.^9^

Our investigations in this area have focused on two distinct yet conceptually related systems that demonstrate the versatility of light as a stimulus in purification systems. In the first, a tetrahedral Zn_4_L_4_ capsule selectively isolates progesterone from steroid mixtures, releasing it upon light-induced cage disassembly.^2^ In the second, a photoresponsive assembly incorporating azobipyridine ligands achieves selective extraction of lithium ions from mixed alkali metal solutions.^4^ Together, these studies highlight how light-triggered cage opening can be harnessed to achieve chemical separations, transforming passive host–guest systems into active molecular filters that operate reversibly, efficiently, and without chemical waste.

### Light-Controlled Molecular Separation of Progesterone

The separation of steroidal compounds from complex mixtures remains a demanding and resource-intensive task, typically requiring multistep chromatographic purification and extensive solvent use.^41–43^ To explore a cleaner alternative, we developed a light-responsive tetrahedral capsule that combines molecular recognition with light-controlled guest release under mild conditions. This system employs Zn_4_L_4_ capsule **2**, assembled by subcomponent self-assembly from the same azobenzene-containing aniline incorporated into cage **1**, a tris­(formylpyridine)­triazatruxene subcomponent, and Zn^II^ ions (Figure 3a). Unlike cage **1**, which binds only small anions, the triazatruxene core expands the internal cavity of capsule **2** relative to that of **1**, allowing it to bind bulkier and neutral steroid guests. As in cage **1**, capsule **2** adopts a stable tetrahedral framework in its *trans* configuration, enabling the encapsulation of hydrophobic guests.

**Figure 3:**
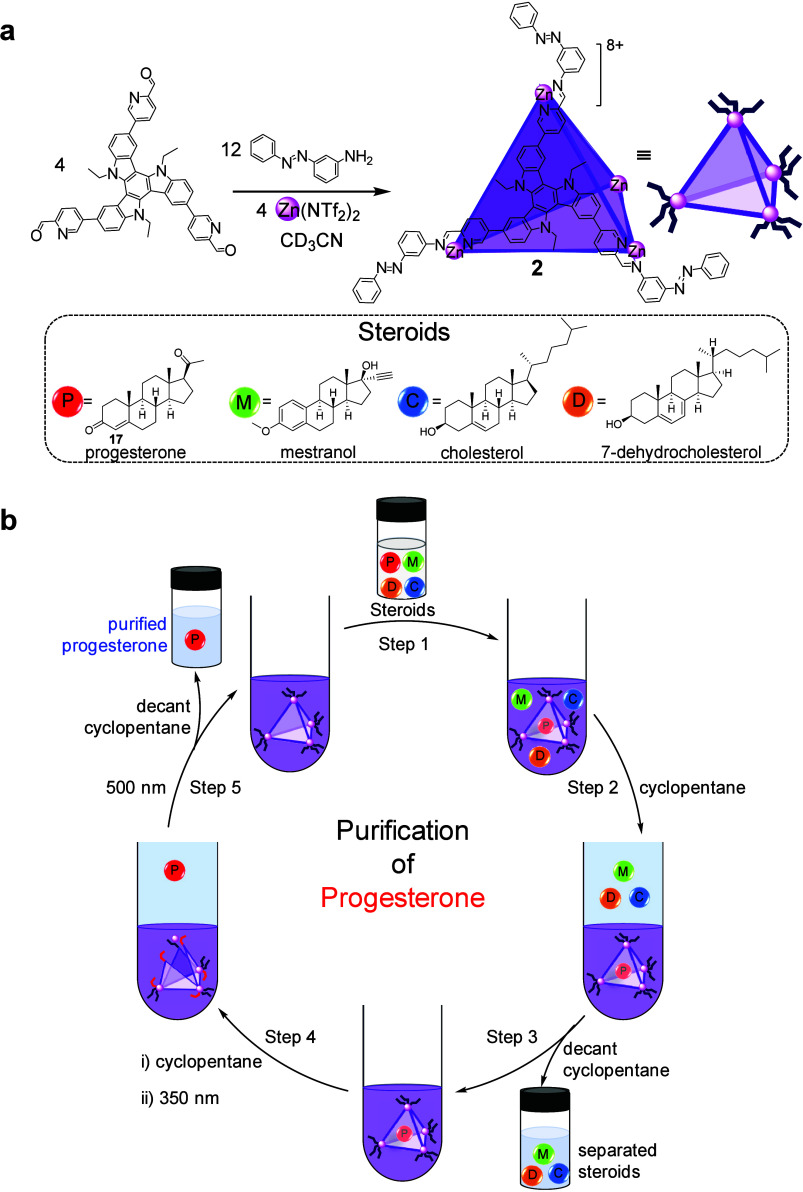
(a) Synthesis of larger cage **2** for steroid encapsulation. (b) Stepwise separation of progesterone using cage **2**, where **2** selectively binds progesterone from a steroid mixture, the other steroids are removed in cyclopentane, irradiation at 350 nm releases progesterone for extraction, and illumination at 500 nm regenerates **2** for reuse.

When an equimolar mixture of progesterone, 7-dehydrocholesterol, cholesterol, and mestranol was introduced, the capsule bound progesterone selectively from solution, while the other steroids remained unbound (Figure 3b). The separation was performed in a biphasic system consisting of acetonitrile and cyclopentane, which facilitated partitioning of free and encapsulated species. This mixture was selected because these steroids partition similarly between the two solvents in the absence of the capsule, making simple extraction ineffective. In the dark, capsule **2** sequestered progesterone within the acetonitrile phase as confirmed by ^1^H NMR spectroscopy. Upon irradiation at 350 nm, the azobenzene units underwent partial *trans*–*cis* isomerization, which disrupted the metal–ligand bonds and released the encapsulated progesterone into the cyclopentane phase. Illumination at 500 nm reversed the isomerization, restoring the *trans* form and reassembling the capsule, which could then bind progesterone once again. We note that this extraction sequence is intended as a proof-of-concept for light-controlled molecular transport, rather than an optimized solvent-minimizing separation method.

This reversible operation enabled up to 78% recovery of progesterone across successive extraction cycles, without detectable decomposition or fatigue of the capsule. Slice-selective ^1^H NMR measurements were employed to monitor the switching process and confirm guest transfer between the acetonitrile and cyclopentane phases. The sequence of experiments demonstrates that light can regulate selective encapsulation and release in solution, offering a controllable approach to molecular separation. Overall, the study illustrates how a photoresponsive capsule can serve as a functional purification unit, capable of distinguishing between closely related steroidal molecules through cavity size and shape complementarity while engaging in reversible, light-mediated binding.

### Selective Lithium Extraction Using Azobipyridine Ligands

The recovery of lithium from complex alkali-metal mixtures represents an important goal in sustainable-energy chemistry, yet selective separation of Li^+^ from Na^+^ and K^+^ remains challenging because of their similar charge densities and coordination preferences.^44,45^ To develop a controllable and energy-efficient alternative, we designed a light-responsive coordination system in which photoisomerization modulates ion binding and release under mild conditions. Building on the design principles established for azobenzene-based cages **1** and **2**, we employed azobipyridine ligands in the present system, which assemble with lithium ions to form the Li_5_L_2_ sandwich complex **3** (Figure 4a). Two planar ligands stack to enclose five Li^+^ ions between them, producing a compact coordination pocket that matches the small ionic radius of lithium while disfavoring larger alkali ions. In the *trans* configuration, the azobipyridine units adopt an extended geometry that aligns donor sites for optimal Li^+^ chelation. The structure shown for **3** (Figure 4a) reflects its solution-state characterization by ^1^H and ^7^Li NMR, DOSY, and ESI-MS. Upon 350 nm irradiation, partial *trans*–*cis* isomerization bends the ligands and perturbs their coordination vectors, weakening Li^+^ binding and promoting release. Visible-light illumination restores the *trans* form, re-establishing the binding geometry and enabling reuptake (Figure 4b).

**Figure 4:**
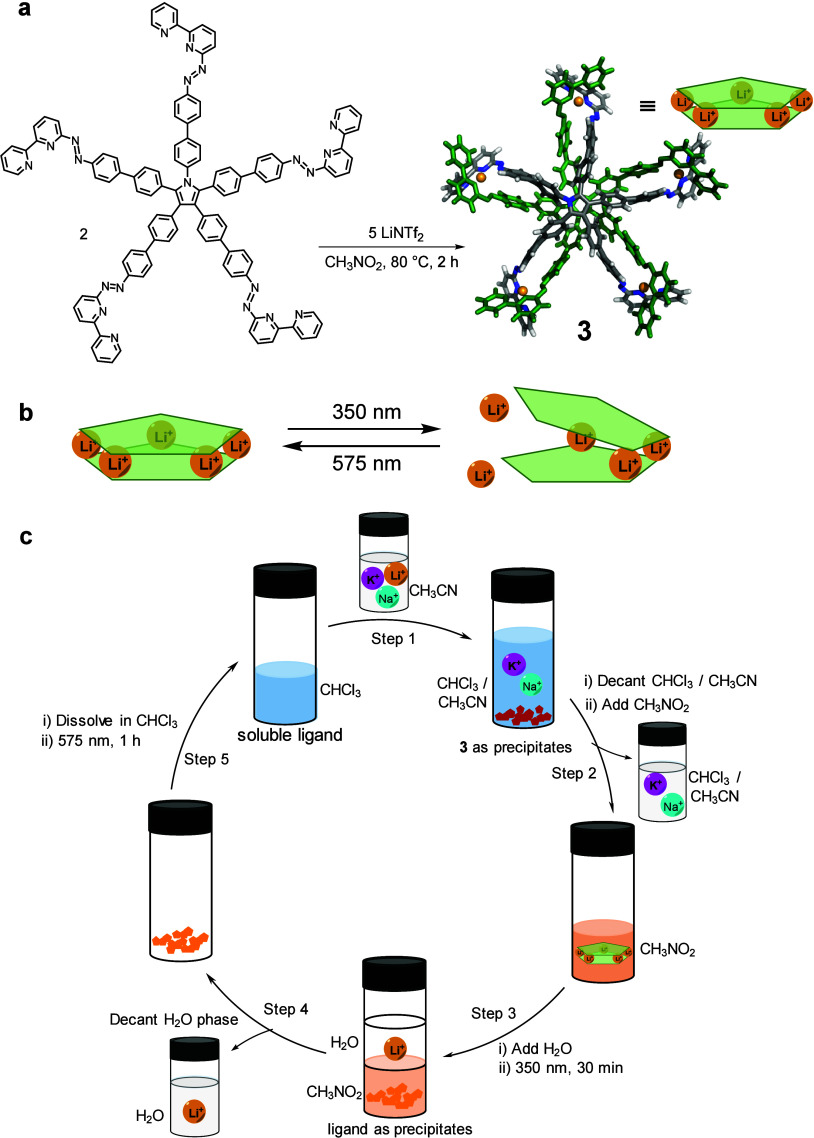
(a) Synthesis and DFT-optimized structure of Li_5_L_2_ sandwich **3**, with one ligand highlighted in green for clarity. (b) Cartoon illustrating the photoswitching of **3** and subsequent Li^+^ release. (c) Lithium ion extraction from a Li^+^/K^+^/Na^+^ mixture using a photoresponsive ligand, featuring selective Li^+^ binding and release, with ligand recycling.

This reversible isomerization allows the assembly to function as a light-gated extractor that pulls Li^+^ from mixed alkali solutions (Figure 4c). Complex **3** forms selectively with Li^+^, and when the precipitate in Figure 4c is redissolved, it regenerates the same spectroscopic signatures, indicating that it contains the same Li_5_L_2_ species, although its solid-state structure was not determined; moreover, its composition can be modulated photochemically. The process proceeds cleanly through multiple cycles with no detectable ligand degradation, highlighting that photoisomerization rather than chemical alteration controls ion release. Overall, the study demonstrates that azobipyridine ligands can couple reversible structural change with selective metal-ion coordination, providing a foundation for light-driven, programmable ion separations relevant to sustainable lithium recovery and battery-material purification.

Together, these two systems show how the design of light-responsive metal–organic architectures can extend beyond molecular recognition to achieve controllable, reversible separations of both neutral species and ions. By integrating photoswitches into self-assembled frameworks, purification processes can be regulated by light, providing a model for future development of energy-efficient and recyclable supramolecular separation technologies.

## Photoswitchable Catalysis

Light-controlled catalysis offers a direct means to modulate chemical reactivity without chemical additives or continuous thermal input.^46,47^ By combining photoswitchable ligands with self-assembled hosts, it becomes possible to confine catalysts within dynamic environments whose accessibility and activity can be toggled on demand.^48^

Capsule **4** was assembled by subcomponent self-assembly of azobenzene-containing amines, tris­(formylpyridine) subcomponents, and Cd^II^ ions (Figure 5a).^3^ Its cavity accommodates a perrhenate (ReO_4_^–^) guest that acts as a precatalyst for the reduction of aromatic aldehydes. In the stable *trans* configuration, the azobenzene ligands preserve the tetrahedral framework, confining the ReO_4_^–^ and suppressing its activity. Irradiation at 350 nm switches the azobenzene units to their *cis* form, perturbing coordination at cadmium and releasing the guest. The liberated ReO_4_^–^ catalyzes aldehyde reduction upon heating at 75 °C, as confirmed by ^1^H NMR monitoring of substrate consumption and product formation. Illumination at 500 nm or thermal relaxation restores the *trans* configuration, allowing the capsule to reform and re-encapsulate perrhenate, switching off the catalytic activity. This on–off behavior is cleanly reversible over multiple cycles with minimal fatigue. Control experiments confirmed that catalysis proceeds only when the cage is open, verifying that the host functions as a reversible gate governing catalyst accessibility.

**Figure 5:**
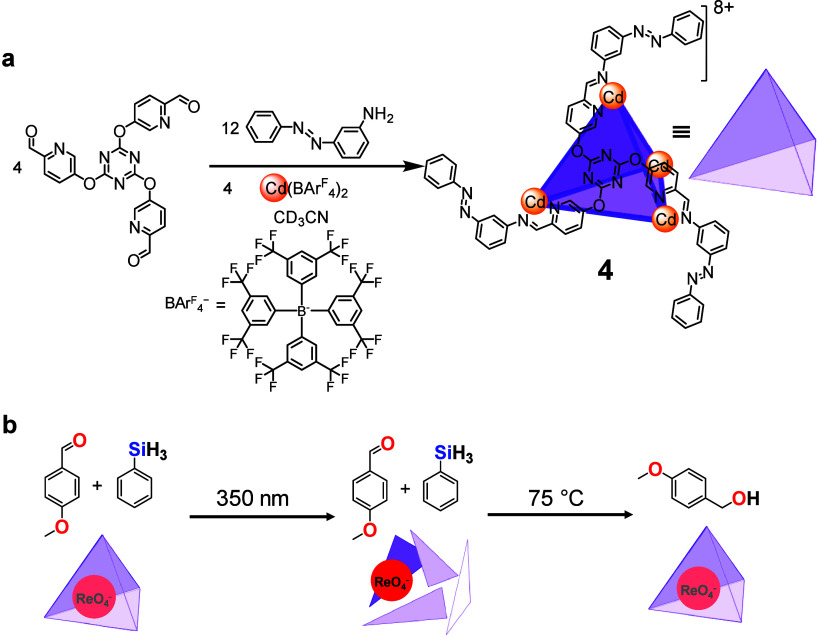
(a) Synthesis of Cd_4_L_4_ cage **4** and its cartoon depiction. (b) Photoswitchable ReO_4_^–^-mediated reduction of *p*-anisaldehyde by cage **4**.

Capsule **4** differs from capsule **1** used to establish key principles of guest release.^1^ In capsule **1**, the counteranion (Tf_2_N^–^) occupies the cavity during assembly, meaning that when a new anionic guest such as ReO_4_^–^ is introduced, an excess of guest must be added to displace the bound anion, preventing a clean “off” process upon reassembly. In capsule **4**, this limitation was overcome by employing the bulky, weakly coordinating [BArF_4_]^−^ anion during assembly. Together with capsules **1** and **2**, this system illustrates how cavity occupancy and anion choice govern the outcome of photoisomerization. This anion is too large to occupy the internal cavity, yielding an empty host that can encapsulate and release ReO_4_^–^ reversibly. The rate and extent of catalysis depend directly on the duration of UV exposure (Figure 5b). Short irradiation produces partial cage opening and limited product formation, whereas longer illumination increases the fraction of *cis*-azobenzenes, promoting greater catalyst release and faster substrate conversion. By varying the irradiation time, product yield can be tuned, establishing a quantitative relationship between optical input and catalytic response.

This system thus demonstrates how photoresponsive metal–organic capsules can serve as dynamic nanoreactors whose activity is externally regulated by light. By coupling reversible structural transformation with encapsulation, catalytic processes can be modulated in a spatially and temporally defined manner, providing a blueprint for light-controlled reaction networks and adaptive chemical systems.

### Transition to Light-Driven Directional Transport

The preceding studies demonstrate how light-responsive metal–organic capsules can reversibly control molecular functions such as guest release, selective separation, and catalytic activation using photoisomerization. Having established this ability to toggle binding and reactivity in homogeneous solution, our next goal was to extend light control beyond local molecular events to directional processes operating across macroscopic distances.^49,50^ By integrating coordination cages into membranes, we sought to harness photoinduced structural change to generate concentration gradients and achieve directional molecular motion, thereby transforming reversible switching into continuous light-driven transport on the macroscopic scale. This progression shows how principles developed for discrete cages may be extended to enable the design of systems that achieve directional transport.

## Light-Driven Directional Transport Across Membranes

The systems described above demonstrate that light can reversibly regulate molecular functions in solution by controlling binding and reactivity within metal–organic capsules.^1–4^ Having established these mechanisms in homogeneous environments, we next sought to extend light control to spatially resolved processes, where molecular motion is guided across interfaces.^51^ We thus developed a supramolecular transport system in which photoisomerization of a guest molecule drives directional flow through an aqueous layer containing coordination cages, in a process mimicking the “Maxwell’s Demon” thought experiment.^52^

The experimental setup consisted of a glass U-tube with two dodecane layers connected and separated by a central aqueous layer containing Fe_4_L_6_ cage **5** (Figure 6a).^5^ The cages in the aqueous phase act as transport mediators, reversibly binding hydrophobic guests and enabling exchange between the two organic compartments. The photoswitchable guest o-tetrafluoroazobenzene (**FAB**) was introduced as the active species (Figure 6b). Its *trans* and *cis* isomers display different kinetics of binding for cage **5**, with the *cis* isomer binding more rapidly than the *trans* form. Illumination of one arm of the U-tube with selected wavelengths of light produced distinct local photostationary states, one enriched in ***cis*** **-FAB** and the other in ***trans*** **-FAB**. The resulting asymmetry generated a net chemical potential difference between the two sides of the aqueous layer, as ***cis*** **-FAB** was transported more rapidly than the *trans* isomer. ***Cis*** **-FAB** was thus transported through the aqueous phase and released into the opposite dodecane arm, where illumination resulted in its isomerization into the more slowly transported *trans* isomer. Sustained asymmetric illumination maintained this cycle, leading to a steady-state concentration gradient of **FAB** across the two arms of the U-tube.

**Figure 6:**
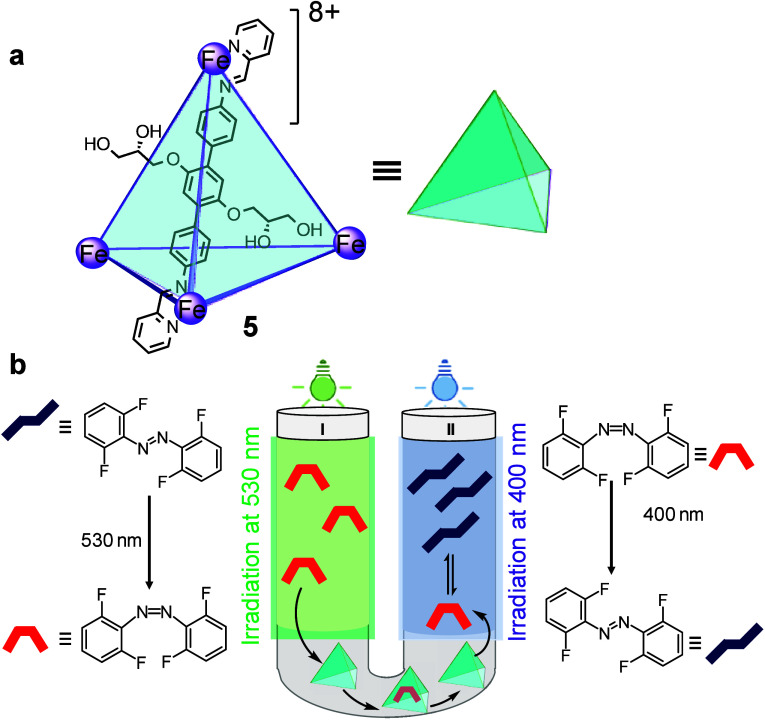
(a) Structure of cage **5**, highlighting one of the six ligands forming the tetrahedron edges. (b) Light-driven preferential transport of a *cis*-azobenzene through an aqueous membrane containing a coordination cage in a U-tube setup, thus establishing a concentration gradient.

Coupled motion of a second, nonswitching molecule, naphthalene, further demonstrated that the system performs chemical work. When naphthalene was added as a co-guest, it was transported in the opposite direction than the **FAB**.This back-transport arises as a result of the different binding kinetics of naphthalene versus the two isomers of **FAB**, with each isomer predominating under different conditions. Naphthalene competes more effectively with ***trans*** **-FAB**, leading to bulk naphthalene transport in the opposite direction to bulk **FAB** transport. When the light source was removed or the illumination made uniform, both gradients dissipated and equilibrium was restored, confirming that the process is reversible and fully dependent on differential illumination. Monitoring by ^1^H NMR and UV–visible spectroscopy showed that transport correlates directly with changes in the local *cis*–*trans* ratio of **FAB**, verifying that the direction and magnitude of molecular flow are governed by the photochemical switching of the guest.

This study demonstrates that coordination cages can mediate light-driven mass transport across macroscopic distances by exploiting the reversible photoisomerization of encapsulated molecules. By coupling molecular switching to concentration gradients, these assemblies convert light input into directional molecular motion, coupling the behavior of discrete supramolecular hosts to emergent nonequilibrium phenomena that define molecular machines and adaptive materials.

## Conclusions and Perspectives

Light-responsive metal–organic capsules thus offer a versatile and modular platform for controlling chemical processes with spatial and temporal precision.^33^ By integrating azobenzene-based ligands into tetrahedral and sandwich architectures, these systems translate light inputs into structural and functional outcomes, including guest encapsulation and release, selective separations, catalysis, ion extraction, and directional transport.^1–5^ This design strategy demonstrates that simple molecular switches can be amplified within self-assembled architectures to perform complex tasks under mild conditions.

Beyond demonstrating functional diversity, these studies reveal key design principles: the position and number of photoresponsive units dictate responsiveness, host–guest interactions can be tuned for selectivity, and light-triggered conformational changes can be harnessed to move material across membranes. Together, these findings lay the groundwork for designing supramolecular systems that translate external stimuli into precise, programmable chemical functions.

Future developments may include: (i) expanding cage topologies to increase guest size or functional diversity,^53–55^ (ii) tuning absorption properties for near-infrared light activation,^56–58^ as is necessary to penetrate living tissue, (iii) integrating multistimuli responsiveness for sequential or logic-based operations,^59,60^ and (iv) combining these capsules with hybrid or polymeric materials to enhance stability and solubility in aqueous, biologically relevant environments.^61–63^ Applications in chemical purification, resource recovery, catalysis, targeted transport, and drug delivery could benefit from these improvements.

Finally, translating light-responsive capsule design into practical technologies requires addressing challenges such as photofatigue, long-term stability, and scalability. Advances in ligand engineering, cage encapsulation strategies, and material hybridization appear to be good routes to overcoming these barriers. In summary, light-controlled metal–organic capsules exemplify how molecular-level design can enable programmable, reversible, and economical chemical functions, paving the way for the next generation of responsive supramolecular systems.
